# The Possible Future Roles for iPSC-Derived Therapy for Autoimmune Diseases

**DOI:** 10.3390/jcm4061193

**Published:** 2015-05-28

**Authors:** Meilyn Hew, Kevin O’Connor, Michael J. Edel, Michaela Lucas

**Affiliations:** 1 Department of Clinical Immunology, Pathwest Laboratory Medicine, Queen Elizabeth II Medical Centre, Perth 6009, Western Australia, Australia; E-Mail: Michaela.Lucas@health.wa.gov.au; 2 Department of Clinical Immunology, Royal Perth Hospital, Perth 6000, Western Australia, Australia; E-Mail: Kevin.O’Connor@health.wa.gov.au; 3 Control of Pluripotency Laboratory, Department of Physiological Sciences I, Faculty of Medicine, University of Barcelona, Hospital Clinic, Casanova 143, Barcelona 08036, Spain; E-Mail: Edel.Michael@gmail.com; 4 Victor Chang Cardiac Research Institute, Sydney, 2010, New South Wales, Australia; 5 School of Medicine and Pharmacology, Anatomy, Physiology and Human Biology, CCTRM, University of Western Australia, Perth, 6009, Western Australia, Australia; 6 School of Medicine and Pharmacology and School of Pathology and Laboratory Medicine, The University of Western Australia, Harry Perkins Institute of Medical Research, Perth, 6009, Western Australia, Australia; 7 Institute for Immunology and Infectious Diseases, Murdoch University, Perth, 6150, Western Australia

**Keywords:** inducible, pluripotent, stem cells, autoimmunity, therapy, lupus, diabetes, multiple sclerosis

## Abstract

The ability to generate inducible pluripotent stem cells (iPSCs) and the potential for their use in treatment of human disease is of immense interest. Autoimmune diseases, with their limited treatment choices are a potential target for the clinical application of stem cell and iPSC technology. IPSCs provide three potential ways of treating autoimmune disease; (i) providing pure replacement of lost cells (immuno-reconstitution); (ii) through immune-modulation of the disease process *in vivo*; and (iii) for the purposes of disease modeling *in vitro*. In this review, we will use examples of systemic, system-specific and organ-specific autoimmunity to explore the potential applications of iPSCs for treatment of autoimmune diseases and review the evidence of iPSC technology in auto-immunity to date.

## 1. Introduction 

Pluripotent stem cells have the ability to differentiate into all three of the embryonic germ layers, endoderm, mesoderm, or ectoderm. While these pluripotent cells may be of embryonic origin, somatic cells can be induced into this pluripotency state by transient ectopic expression of defined groups of transcription factors, hence the term “inducible” pluripotent stem cells (iPSCs). The advantages of inducing pluripotency includes the potential generation of unlimited numbers of required cells, deriving cells from hard-to-source tissues, reproduction of disease models, bypassing the ethical concerns regarding the use of embryonic stem cells and importantly provide an autologous cell therapy strategy that removes the need for immune suppression drugs.

## 2. Background

Following the seminal paper by Takahashi and Yamanaka [1], which reported using appropriate transcription factors, Oct4, Sox2, Klf4, and c-Myc, mouse fibroblasts could be reprogrammed into a pluripotent state, it has been demonstrated in human somatic cells. Furthermore, other combinations of transcription factors are able to induce pluripotency in human somatic cells as well [1,2,3,4,5].

Autoimmune diseases affect individual organs or a combination of organs, including the kidneys, brain, bone marrow, joints, or skin, however, the pathogenesis of most autoimmune diseases remains, at best, only partially delineated. IPSC technology has the potential to provide key cellular subsets which, given to patients, may alter their disease course by providing pure replacement of lost cells, may limit damage through immune-modulation of the disease process *in vivo*, and may provide substrates for the purposes of disease modeling *in vitro*. In this review, systemic lupus erythematosus (SLE) is taken as a prototypical example of a systemic auto-immune disease, along with rheumatoid arthritis (RA); diabetes mellitus (DM) as an example of organ specific autoimmunity; and multiple sclerosis (MS) as an example of system-specific neurological autoimmunity, to demonstrate the promising future research potential towards translational medicine of iPSC-derived treatment in a range of different contexts within Clinical Immunology.

## 3. Disease Immunomodulation and Potential Cellular Components—SLE and RA as Examples

The loss of tolerance to self is the fundamental basis of autoimmunity, with resultant aberrant immune responses of autoantibody formation and/or cellular immunity against self-tissue.

Systemic lupus erythematosus (SLE) is a prototypical systemic autoimmune disease. Usually affecting women of childbearing age, it is characterised by the production of multiple auto-antibodies directed against double-stranded DNA and other nuclear antigens, which are widely distributed throughout the body. The autoantibodies are produced by activated auto-reactive B cells following presentation of these self-antigens to self-reactive T cells. Along with autoantibody production are reduced populations of regulatory T cells (Tregs), reduced responses to regulation by these cells on effector T cells, immunological dysregulation and increased inflammation [6], immune complex formation and deposition, and end-organ damage, particularly if the disease affects the kidneys or central nervous system.

Rheumatoid arthritis is a symmetrical, inflammatory disease of synovial joints which also manifests extra-articular pathology in about 40% of patients. Affecting other parts of the musculoskeletal system, as well as the skin, eye, lung, heart, kidney, and vascular and nervous system tissues, it is likely that the inflammatory processes driving the synovial inflammation are also responsible for these extra-articular manifestations. RA patients develop autoantibodies to post-translationally modified synovial or stress-related proteins, which results in the conversion of arginine residues into citrulline (a process known as citrullination). In genetically susceptible individuals, preferential binding of these citrullinated self-peptides to MHC molecules may enable presentation to peripheral T cells, allowing expansion of potentially self-reactive T-cell populations. At the same time, if there is no presentation centrally in the thymus, there is no deletion or negative selection of autoreactive T cell populations, which is a possible mechanism for loss of self-tolerance in RA.

The mainstay of treatment, for both SLE and RA, is with immunosuppressive medications, however, true immunomodulation in the absence of toxicity is difficult to achieve.

There are a number of important cell populations that impact on systemic autoimmune disease course in which iPSC technology could potentially assist to model their effects and ideally contribute to regaining self-tolerance, such as regulatory T cells (Tregs) and dendritic cells. Targeting of particular cell lineages, rather than their end products, is also likely to be beneficial in the treatment of other autoimmunity diseases.

### 3.1. Regulatory T Cells (Tregs)

Regulatory T cells (Tregs), have an important role in the state of equilibrium that is immune tolerance, and are, therefore, also known as tolerogenic T cells. Tregs are CD4, CD25, and Foxp3 positive, and act to restrict the extent and duration of T cell mediated immune responses, and maintain peripheral tolerance by suppressing auto-reactive T cells that have escaped negative selection in the thymus. The mechanisms by which Tregs work continue to be discovered [7]. Most Tregs arise centrally in the thymus where cell lineage commitment is determined by T-cell receptor (TCR) specificity to self antigen. The transcription factor Foxp3 stabilises gene expression that specifies Treg differentiation while other transcription factors, including c-Rel, links TCR engagement and Foxp3 expression, within an appropriate cytokine and co-stimulatory molecule milieu, for Treg differentiation.

In the periphery, Tregs can be induced following repeated antigen exposure [8] under the influence of TGF-beta, converting Foxp3 negative T cells into Foxp3 positive induced Tregs (iTregs). Hence, this replaces T effector populations with regulatory populations, converting harmful responses to beneficial regulatory responses.

The list of potential defects in Tregs leading to autoimmune diseases are many (Table 1). Considering this extensive list, however, enables multiple potential targets for iPSC application and analysis of disease processes.

**Table 1:** Potential defects in regulatory T cells in autoimmune diseases [9,10].

Imbalances in peripheral effector and regulatory T cells due to defects in thymic selection Genetic defects inducing failed Treg function or inadequate Treg activity Overwhelming of Treg responses due to epitope spreading in autoimmune diseases, Deficient IL-2 (required for Treg development) Low CD25 expression (hence reduction of IL-2 signalling) Defective conversion of naive T cells to adaptive Tregs (due to IL-10 or TGF-beta deficiency) APC maturation defects leading to altered T cell activation and altered development of tolerogenic phenotype Hyper-costimulation by APCs leading to pathogenic T cells rather than tolerogenic phenotype Aberrant cytokine milieu leading to Treg suppression

The transfer of autologous Tregs to suppress immune responses has already been demonstrated experimentally in SLE and other autoimmune diseases such as diabetes mellitus [11,12]. Regulatory T cells are present at locations of inflammation (e.g., synovial fluid, mucosa) [13] though, if regulatory T cells are obtained from these sites, there may be inadvertent contamination of auto-reactive effector T cells, which could lead to unintended inflammatory consequences from therapeutic reinfusion of collected cells. Once isolated, it is technically challenging to induce these regulatory T cells to proliferate exogenously, which places limits on the application of harvested Tregs from patients for use in therapeutic treatments.

The ability to instead induce functional Tregs rather than needing to collect them, has been demonstrated from iPSCs *in vivo* [14]. These cells produced the immunoregulatory cytokines TGF beta and IL-10, thus producing a population of presumably functional Tregs. In a promising find, both allogeneic and autologous transfers of these iPSC derived Tregs demonstrated clinical efficacy, by reducing disease incidence and clinical severity scores in collagen-induced arthritis (CIA), an inducible mouse model of RA.

### 3.2. Dendritic Cells

Dendritic cells are highly proficient APCs that are potent in stimulating naive T cells during the primary immune response [15]. Numerous abnormalities in dendritic cells have been noted in patients with autoimmune diseases, including variations in cells proportions, differences in cytokine receptor expression particularly inhibitory receptors, and increased expression of costimulatory molecules [16,17].

Conventional dendritic cells (cDCs, previously known as myeloid DCs) are extremely efficient APCs, expressing several Toll-like receptors (TLRs) on their surface and producing TNF-alpha, IL-1, IL-6, IL-12, and IL-10 upon stimulation. Under different stimuli, cDCs can demonstrate different tolerogenic phenotypes, inducing antigen-specific unresponsiveness in central and peripheral lymphoid organs, and, therefore, have a crucial role in the induction of immune tolerance [18]. These tolerogenic dendritic cells are characteristically able to induce proliferation of Tregs (which then modulate immune responses to self-antigens), and to induce anergy in auto-reactive effector T cells [18,19]. Depending on the stimuli applied to the cDCs, different tolerogenic phenotypes are demonstrated, with functional differences in the Treg responses that are elicited [10]. Thus, depending on the desired Treg outcome, there is potential to preferentially select these outcomes by altering the particular phenotype of the applied tolerogenic dendritic cell in disease immunotherapy.

For example, Tregs can be induced *in vivo* by NFKB or CD40-deficient DCs. Conventional DCs require the transcription factor RelB to enable priming of the immune system through CD40 and MHC-molecule expression [20,21]. Blocking of RelB and other NFKB family members in cDCs results in induction of Tregs through modified cDC activity, therefore RelB activity is thought to determine the outcomes of antigen-presentation to cDCs. Methods to block RelB activity, and that of other NFKB family members have been developed to produce modified DCs that are consistently tolerogenic through the induction of Tregs [20,22,23]. In murine models of antigen-induced arthritis, modified DCs have been shown to suppress joint inflammation and erosion [24]. As tolerance induction by these DCs has been shown to be dose-dependent and route-independent [22], after induction of inflammatory arthritis by joint injection of methylated bovine serum albumin (mBSA), the mice were able to be subcutaneously injected with modified DCs exposed to mBSA, resulting in a suppression of inflammatory responses in the joints.

Given proof of concept studies using regulatory DCs in immunotherapy have demonstrated a reduction in effector T cell in other autoimmune diseases [25,26] the use of regulatory DCs as autologous immunotherapy is an exciting focus for possible future therapies [10,16,17], particularly in the immunomodulation of the inflammation noted in SLE and RA.

Plasmacytoid dendritic cells (pDCs) constitutively produce anti-viral Type 1 interferons as part of the immune response to viral infections. However, in patients with autoimmune diseases, such as SLE, pDCs are thought to instead make interferons following TLR ligation by endogenously derived nucleic acids [27]. The immune response is, thus, driven not by exogenous infection, but by activity against self-antigens.

Plasmacytoid dendritic cells that produce Type I interferons are found in the tissues of affected organs in SLE and other autoimmune conditions. Type I interferons have activity through several down-stream pathways to increase dendritic cell maturation and activation and, hence, antigen presentation to immune lymphocytes, and non-haematopoietic cell cytokine and MHC expression [6]. This immune activation results in up-regulated inflammation, and a positive-feedback loop with further dendritic cell production of interferon, and resultant anti-self T cell activation and B cell auto-antibody production.

In patients with active SLE, polymorphonuclear lymphocytes (PMNLs) have been shown to up-regulate interferon genes giving an interferon “signature”, which correlates with disease severity, and high dose steroids which abrogate this signature induce clinical remission. Depletion of pDCs early in the course of SLE can reduce the clinical and serological evidence for autoimmunity [28]. This evidence indicates that the ability to model the interactions of pDCs would be beneficial to understanding more of the underlying pathogenesis in SLE.

The routine use of dendritic cells for research into the generation of immunomodulation, or for disease modeling *in vitro*, in SLE, RA and other autoimmune diseases is limited by the lack of plentiful and stable dendritic cells of the appropriate phenotype. Peripheral collection of precursors for autologous transfer through plasma exchange is not without morbidity, and the cost and logistics for wide-spread collection may not be feasible. Therefore, while able to be generated from haematopoietic stem cells, regulatory dendritic cells have recently been generated from murine iPSCs [19]. These iPSC-derived regulatory dendritic cells have been shown to have similar morphology to bone marrow derived regulatory DCs, and appeared to have similar activity to bone marrow derived regulatory DCs in not stimulating allogeneic CD4+ T cells, only weakly stimulating allogeneic CD8+ T cells and having similar efficient antigen uptake. What remains is to demonstrate stable phenotype and function, which can then enable comparison of results in clinical trials and other applications to be explored.

Once cells are generated from iPSCs, these need to have a valid functional assessment for tolerogenic properties. Similarly, as there is a theoretical risk for replication of the disease process with autologous transfer of cells, and a demonstrated risk for malignancy with iPSCs, appropriate monitoring and assessments will be required.

### 3.3. Disease Modelling in SLE or RA

Theoretically, the potential for disease modelling could be greatly expanded by generating and studying the different tissue lineages from patient-derived iPSCs [3]. While neurological tissue collection remains elusive, methods for expansion of renal specific cells into iPSCs through non-invasive urinary cell collection has been described [29]. Therefore *in vitro* examination of pathological processes using iPSCs derived from affected patients, and, possibly, regeneration of tissue from unaffected patients may both be possible. However, the end-organ damage of SLE is a manifestation of systemic immune dysregulation therefore the targets of therapy or investigation may be more well-focussed on the interactions between cellular populations and an examination of the matrix of effects on tolerance and auto-reactivity. Both SLE and RA are multifactorial in their pathogenesis with a complex interaction between environment and genetics, resulting in the loss of self-tolerance [30,31].

## 4. Generation of Reparative Tissue in Autoimmunity—Diabetes Mellitus

Diabetes mellitus is a significant clinical problem with high morbidity and mortality associated with microvascular and macrovascular complications of hyperglycaemia. Arising either from beta cell dysfunction and insulin resistance, or from autoimmune cell-mediated pancreatic islet cell destruction and resultant lack of insulin, treatments are usually aimed at glycaemic control, or reducing insulin resistance. Accurately and consistently replacing insulin at an amount appropriate for associated oral intake can be difficult for patients, with the risk for unstable sugars and hypoglycaemia.

Replacement of pancreatic tissue through tissue donation is in current use, however limited through lack of donors and restrictive through the requirement for life-long immunosuppression. It has been previously pointed out therefore, that treatment for diabetes would ideally renew beta cell function and, hence, insulin for glycaemic control, prevent repeat autoimmune destruction of the new pancreatic tissue, and repair the micro- and macrovascular complications that may have already occurred [32].

The current state of play with iPSCs and diabetes, also detailing concerns of immunogenicity, tumorigenicity, appropriate differentiation, full maturation, stability of function, and successful engraftment have recently been reviewed [33] with much work still required for understanding the basic biology of reprogrammed cells.

However, in terms of current research aspirations, there is great interest in attempting to recapitulate normal pancreatic development and generate pancreatic cell types from pluripotent cells [34]. This would encompass differentiating iPSCs into definitive endoderm, morphogenesis into a three-dimensional structure with contact with appropriate mesenchymal supportive cells to provide required growth and development signals, and then commitment of the pancreatic endoderm to endocrine precursor cells and thence to beta cells that produce the required insulin in a glucose-responsive fashion.

Thereafter, considerations need to be made on prevention of rejection of transplants, potentially preferring patient-specific iPSC generation and autologous transfer [35]. iPSC lines have so far been generated from patients with type 1 and type 2 diabetes, as well as maturity-onset diabetes of the young [36,37,38].

In terms of functional beta cell production, polyhormonal insulin-expressing cells have been derived from human embryonic stem cells and transplanted for some years now, though whether from insufficient cell volume transfer, or transfer of functionally immature beta cells, while helping fasted blood glucose states, they do not yet consistently ameliorate diabetes in non-fasted mice subjects, or tend to lose insulin-secretion capacity [39,40,41]. In an alternative line of investigation, when given enough time to develop *in vivo* (90–140 days post transplant), engraftment of pancreatic progenitor cells derived from human embryonic stem cells have been able to secrete insulin, and maintain normoglycaemia in a murine model of induced diabetes up until the grafts are removed [42].

Subsequently, glucose-responsive, insulin-producing cells have been generated from human iPSCs and also shown to have the ability in murine models to reverse hypoglycaemia [43], however, can lose insulin secretion over time [44]. While it is important to remember that there are differences between embryonic stem cells and iPSCs [45], potentially, progenitor pancreatic cells may be developed as well from iPSCs for trials in engraftment, but with the advantages inherent over requiring embryonic cell sources.

## 5. iPSCs in Autoimmune Neurological Disease—Multiple Sclerosis

Inducible pluripotent stem cells have been studied extensively in neurodegenerative and neurogenetic disorders, more so currently than for inflammatory neurological conditions, such as multiple sclerosis (MS), however, the final common pathway of neuronal injury and death is better understood in MS than for neurodegenerative conditions. IPSC technology allows potential avenues for therapeutics by regeneration of specific neuronal populations [46] or for exerting an immunomodulatory effect [47], but also allowing more accurate modelling of neurological disease than can be obtained through animal studies [46].

MS is the archetypal and most common disabling autoimmune condition of the central nervous system (CNS), which provides an ideal framework for research and understanding immune dysregulation. MS is a chronic condition, characterised by focal or multifocal inflammatory demyelinating episodes resulting in neurological disability depending on the area of the CNS involved. There are periods of quiescence and recovery in the most common phenotype, known as remitting relapsing MS [48].

The pathogenesis of MS and its triggers are multi-factorial with a complex interaction between genetic predisposition and environmental factors resulting in immune dysregulation. The first risk allele to be identified was the human leukocyte antigen (HLA) class II haplotype HLA-DRB*1501 in the 1970s [49]. The Genome-wide Association Study (GWAS) has since identified over 50 susceptibility loci [50], many of which encode for pro-inflammatory IL-2 and IL-7 [51], with others encoding for cytokines, such as CXCR5, IL-12A, IL-12β, and IL-12Rβ1 [48].

The genetic association alone does not explain fully the development of MS with vitamin D3 and Epstein-Barr virus (EBV) both being important environmental factors to consider in MS. Increased latitude is associated with lower serum levels of vitamin D3, due to lower levels of sun exposure, which corresponds with the higher incidence and prevalence of MS in these high latitude countries [48,52] though the effect of vitamin D3 deficiency on adaptive immunity is not yet fully understood. What has also been observed, is that individuals who are seronegative for EBV have almost no risk of developing MS [53], and it has been hypothesised that, through molecular mimicry, EBV may mimic myelin basic protein pathogenic antigens by presentation on HLA-DRB1*1501, therefore, providing links to both environmental and genetic risk factors [48,54]. Myelin reactive CD4+ T cells secreting interferon gamma are one of many T cell mediators in the pathogenesis of MS [55], with the role of other cell types and cell subsets being also involved, with a reduction in effector function of Tregs in MS patients [56], and a key role of pro-inflammatory T helper 17 (Th17) cells emerging [48,57]. Given the production of oligoclonal bands in CSF, there is a role of B cells in MS pathology, and the understanding of the part played by innate immunity by way of NK (natural killer) cells and dendritic cells in the pathogenesis is evolving [48].

Given the significant effects of MS on affected patients, efforts to provide regenerative or immunomodulatory therapy are highly sought.

Oligodendrocyte precursor cells (OPCs) derived from iPSCs, first described by Onorati *et al*. in 2010 [58], possibly provide an exogenous way in which to remyelinate axons as soon as possible after an episode of acute demyelination, to best protect axons from ongoing inflammation and eventual gliosis. Axonal loss is responsible for the most debilitating functional deficits in the more progressed stages of MS, with this loss followed by retrograde neuronal degeneration [59]. Axonal degeneration not only occurs in chronic lesions, with good evidence now showing axonal injury in acute lesions [60].

Cell replacement with OPCs derived from iPSCs have been shown to be successful in animal studies, with remyelination and amelioration of disability in experimental autoimmune encephalitis (EAE), an animal model of MS [61,62].

Neural precursor cells (NPCs) derived from iPSCs have also been shown in EAE to not only have a regenerative effect, but also an immunomodulatory effect. One study, in which mouse iPSC-derived NPCs were intrathecally transplanted in mice with EAE, exerted a neuroprotective effect, not by differentiating into myelin producing cells, but by producing the specific neurotrophin, leukaemia inhibitory factor (LIF), which supports the *in vivo* survival and differentiation of native oligodendrocytes [63]. LIF has been shown to inhibit the differentiation of Th17 cells through MAP kinase suppression of the cytokine signalling 3 (SOCS3) inhibitory signalling cascade, antagonising the interleukin 6 (IL-6)-mediated phosphorylation of signal transducer and activator of transcription 3 (STAT3) [64], which is essential for the differentiation of Th17 cells, thus limiting CNS inflammation and hence subsequent tissue damage.

Finally, the disease in a dish approach may give unique insights into the study of pathogenesis in neuronal disease and in particular to inflammatory diseases of the CNS, given its inaccessibility. IPSCs have been successfully derived from a MS patient’s dermal fibroblasts, and differentiated into astrocytes, oligodendrocytes and neurons with a normal karyotype. The patient-derived neurons showed electrophysiological differences compared with the control cell line, paving the way for a novel approach to the study of MS pathogenesis [65].

## 6. Conclusions 

Autoimmune diseases are the result of a combination of environmental influences acting on a susceptible genetic background. This causes significant aberrations of self-antigen recognition, lymphocyte activation and differentiation, production of pro-inflammatory cytokines and autoantibodies, and the final end product of tissue and organ damage. Induced pluripotent stem cells technology has the potential to create new safe treatment options, as well as better models to study disease and therapies *in vitro*. Here, we review the so far limited literature in this field. In addition to organ replacement strategies where iPSC technology has been applied, we propose that complex auto-immune diseases require unique immunomodulatory therapy strategies using cellular components and that these components could be made by iPSC technology. Importantly, iPSC technology enables us to produce, differentiate and genetically modify large numbers of immune cells that can be used therapeutically. Prior to the development of such technologies modification of small cell populations with limited *ex vivo* expansion potential was near impossible. Nevertheless, these novel approaches will need to have extensive functional and safety assessments prior to their use in a clinical setting.

Finally, iPSC technology allows for modelling of normal and diseased (based on genetic and epigenetic modifications) cellular growth and development, influences of mutations onto function and clinical phenotype. In the time of personalized medicine iPSC technologies are likely to feature as a key therapeutic tool in auto-immune diseases.
